# Bedside Ultrasonography-Guided Nasogastric Tube Placement: Scoping Review

**DOI:** 10.3390/healthcare14070859

**Published:** 2026-03-27

**Authors:** Mónica Francisca Santana Apablaza, Mayra Gonçalves Menegueti, Vinicius Batista Santos, Rosana Aparecida Pereira, Priscilla Roberta Silva Rocha, Fernanda Raphael Escobar Gimenes

**Affiliations:** 1 Ribeirão Preto College of Nursing, University of São Paulo, Ribeirão Preto 14040-901, Brazil; mfsantana@usp.br (M.F.S.A.); mayramenegueti@usp.br (M.G.M.); rosanna.pereira@gmail.com (R.A.P.); 2 Paulista School of Nursing, Federal University of São Paulo, São Paulo 13418-900, Brazil; v.santos@unifesp.br; 3 Department of Nursing, University of Brasília, Brasília 70910-900, Brazil; priscillarocha@unb.br

**Keywords:** evidence-based practice, intubation, gastrointestinal, nursing, ultrasonography

## Abstract

**Highlights:**

**What are the main findings?**

- The esophagus and the gastric antrum were the anatomical landmarks most often used during ultrasound-assisted verification of nasogastric tube placement.
- Dynamic “fogging,” usually generated by air insufflation, was commonly used to facilitate interpretation, and some protocols added color Doppler to improve visualization.

**What are the implications of the main findings?**

- The esophagus and gastric antrum appear to be consistent and reproducible sonographic landmarks that could support protocol development and clinical standardization.
- The frequent use of dynamic air insufflation suggests clinical utility, while also underscoring the need for standardized performance and interpretation.

**Abstract:**

**Objectives**: This scoping review synthesized the available evidence on bedside ultrasonography used to confirm short-term nasogastric tube (NGT) placement in adults. **Methods**: The review followed JBI Collaboration methodology. Searches were conducted in CINAHL, Embase, LILACS, PubMed, and Scopus, as well as in gray literature sources (Google Scholar and ProQuest Dissertation & Thesis Global). Primary studies and clinical guidelines addressing bedside ultrasonography for short-term NGT placement in adults (≥18 years) were eligible, with no limits on language or publication year. Data were extracted and narratively summarized with the I-AIM framework (Indication, Acquisition, Interpretation, and Decision-Making). **Results**: Twenty-nine studies met the inclusion criteria. Most were single-center observational studies performed in intensive care units or emergency departments. Ultrasound was primarily used for confirmation prior to enteral nutrition initiation, while gastric decompression was less frequently reported. Acquisition protocols varied, although supine positioning, convex abdominal probes, and linear cervical probes were most commonly described. The gastric antrum and esophagus were the principal anatomical landmarks, with interpretation based on direct tube visualization and dynamic fogging; color Doppler was occasionally used. Radiography remained the reference standard in most studies, and only a minority initiated feeding based solely on ultrasound findings. Reported facilitators included bedside feasibility, absence of radiation exposure, and timeliness. Barriers included operator dependency, limited visualization in patients with obesity or gas interposition, protocol heterogeneity, and the limited methodological robustness of available studies. **Conclusions**: Current evidence suggests that ultrasonography may represent a feasible, radiation-free bedside approach for confirmation of NGT placement. Evidence from selected studies suggests that, with structured training, healthcare professionals may achieve diagnostic accuracy in specific clinical settings, although further robust multicenter investigations are needed to confirm these findings.

## 1. Introduction

Patient safety remains a global concern, with millions of adverse events each year leading to preventable harm and deaths [1]. Among high-risk clinical procedures, confirmation of nasogastric tube (NGT) placement is a common nursing procedure associated with potentially life-threatening complications, including aspiration and pneumothorax [2,3]. Misplacement rates vary from 0.3% to 8% [4], and in the United Kingdom, undetected malposition is classified as a “never event” because of its serious consequences [5].

Traditional bedside verification methods, particularly epigastric auscultation, have been widely employed for decades; however, robust evidence demonstrates that this technique lacks sufficient diagnostic accuracy and reliability. Clinical guidelines explicitly discourage its use as a sole confirmation method [6]. Nevertheless, empirical studies indicate that a substantial proportion of nurses continue to rely on auscultation in routine practice, frequently without full awareness of its documented limitations and associated safety risks [7].

Radiography remains the gold standard, providing accurate confirmation of tube position [8]. However, routine use is not without challenges: repeated exposure to ionizing radiation, delays in feeding or medication administration, and additional costs to health systems [9,10].

Ultrasonography has been proposed as a potential alternative strategy for confirmation of NGT placement. It allows for real-time visualization of the tube during or after insertion, avoids radiation, and may contribute to reducing delays in care [11,12]. For nurses, point-of-care ultrasonography (PoCUS) has been described as a tool that may enable immediate bedside confirmation and support clinical decision-making [13,14]. However, the current body of evidence is predominantly composed of observational and single-center studies, with variability in protocols, operator training, and outcome definitions, which limits generalizability and warrants cautious interpretation of findings. Additionally, uncertainties remain regarding diagnostic accuracy across different clinical contexts and levels of professional experience.

Moreover, existing studies describe heterogeneous ultrasonographic protocols, including differences in anatomical landmarks, probe positioning, timing of assessment, and confirmation criteria [11,12,13,14]. Current clinical guidelines primarily recommend radiographic confirmation and do not provide standardized recommendations for ultrasonographic confirmation of NGT placement [5,8]. This lack of international consensus further complicates implementation in clinical practice.

Ongoing education and competency in NGT insertion and verification are essential to prevent adverse events and to ensure evidence-based nursing care [15,16]. In this context, ultrasonography has been suggested as a potential strategy to enhance safety and professional autonomy in nursing practice, although further robust evidence is needed to consolidate its role.

Despite growing interest in point-of-care ultrasonography, previous scoping and systematic reviews have addressed related aspects of ultrasound use in enteral tube management. These reviews present scope limitations and do not specifically map the practical procedures employed for bedside verification of NGT placement in adult patients. Therefore, a comprehensive synthesis focusing on the procedural aspects of ultrasonographic verification remains warranted. By synthesizing available evidence, the findings can inform nursing education, support the development of standardized protocols, and guide the safe integration of ultrasonography into routine care. Nevertheless, conclusions must be interpreted in light of the methodological heterogeneity and limitations of the existing studies. Despite increasing interest in PoCUS, no review has systematically mapped the procedural aspects and implementation characteristics of bedside ultrasonography for NGT placement in adult patients.

The aim of this scoping review was to explore the procedures used in bedside ultrasonography for confirmation of NGT placement in adults.

## 2. Materials and Methods

### 2.1. Protocol and Registration

This scoping review was conducted following the methodology by the JBI Collaboration [17] and reported following the Preferred Reporting Items for Scoping Reviews (PRISMA-ScR) [18]. The protocol for this review was developed prior to study initiation and can be accessed at: https://osf.io/yw9ga/overview (accessed on 4 November 2025).

### 2.2. Eligibility Criteria

The research question for this scoping review was formulated using PCC framework (Population, Concept, Context) [17]: What procedures are used to confirm a short-term nasogastric tube placement (concept) with the guidance of bedside ultrasonography (context) in adults (population)?

For the purposes of this scoping review, PoCUS was defined as the use of portable ultrasound performed directly by a healthcare provider at the point of care, for real-time diagnostic or clinical monitoring purposes [19]. Nasogastric tube was defined as a short-term feeding device, intended for use up to 4–6 weeks, inserted through the nose and positioned in the stomach [20].

We included primary studies and clinical guidelines that focused on the procedures used for bedside ultrasonography-guided short-term NGT placement in adults (≥18 years). No restrictions were applied regarding publication date or language.

Exclusion criteria included: (1) studies that did not address the research question; (2) studies that investigated the use of PoCUS solely for gastric residual volume (GRV) assessment; (3) studies involving newborns, children, adolescents or animals; (4) reviews, abstracts, letters, expert opinion; study protocols, trial registrations; (5) studies involving nasoenteric tubes or long-term feeding tubes.

### 2.3. Information Sources and Search Strategy

The search was conducted across major health science databases, including CINAHL (via EBSCOhost), Embase (via Elsevier), LILACS (via BVS), PubMed (via National Institutes of Health), and Scopus (via Elsevier). Additional sources consulted included gray literature (Google Scholar and ProQuest Dissertation & Thesis Global), and both national and international guidelines.

The search strategy was developed using a combination of controlled vocabulary (Medical Subject Headings [MeSH] and Health Sciences Descriptors [DeCS]) and free-text keywords. The terms were organized according to the PCC acronym: (P) adult population, (C) nasogastric tube placement, and (C) ultrasonography. Controlled vocabulary was adapted to each database, and free-text terms were searched in titles and abstracts. Boolean operators (AND/OR) were used to combine synonyms within each domain and to link the three concepts. The core search structure was as follows: (“Ultrasonography”[MeSH] OR ultrasonography OR POCUS OR “point-of-care ultrasound”) AND (“Intubation, Gastrointestinal”[MeSH] OR “Nasogastric Intubation” OR “Nasogastric Tube” OR “nasogastric tube placement”) AND (“Adult”[MeSH] OR adult). All searches in electronic databases and gray literature were completed on 31 August 2025 (Tables S1 and S2).

### 2.4. Selection Process

Following the search strategy, all retrieved articles were exported to EndNote^®^ for duplicate removal. The remaining articles were then uploaded to the Rayyan^®^ platform, where two authors independently (M.F.S.A. and M.G.M.) applied the inclusion and exclusion criteria in two stages: initial screening by title and abstract, followed by full-text review. Discrepancies were resolved through consensus, with a third author (F.R.E.G) consulted when necessary.

### 2.5. Data Collection Process and Data Items

Data extraction was conducted collaboratively by two reviewers (M.F.S.A and M.G.M). Extracted data were organized in a Microsoft Excel^®^, where the reviewers mapped the findings, discussed results, and continually updated data collection forms. Consensus was sought for any disagreements.

A descriptive synthesis was undertaken; the collected data included: author(s), year of publication, country of the corresponding author, study design and aim, setting, sample and data about I-AIM framework (Indication, Acquisition, Interpretation, and decision-Making) [21].

### 2.6. Synthesis of Results

Data extracted from the included studies were grouped to synthesize the results descriptively. To enhance interpretation, narrative and tabular presentations were complemented with visual tools (infographics and schematic figures) that highlighted procedural variations, recurring patterns, and gaps in practice.

Generative artificial intelligence (ChatGPT 5.0, OpenAI, San Francisco, CA, USA) was used in this study to support English language revision for clarity and proficiency and to assist in the conceptual organization. The AI tool was not used to generate original data, perform data analysis, interpret results, or draw scientific conclusions. All content was critically reviewed, edited, and validated by the authors, who take full responsibility for the integrity and accuracy of the manuscript.

Critical appraisal of individual sources of evidence was not conducted. In accordance with the JBI Collaboration methodology for scoping reviews [17], formal assessment of methodological quality or risk of bias is not mandatory when the objective is to map the extent, characteristics, and nature of available evidence rather than to evaluate intervention effectiveness. The findings are therefore presented descriptively, and methodological heterogeneity is addressed narratively in the Section 4.

## 3. Results

### 3.1. Selection of Sources of Evidence

**Figure 1:**
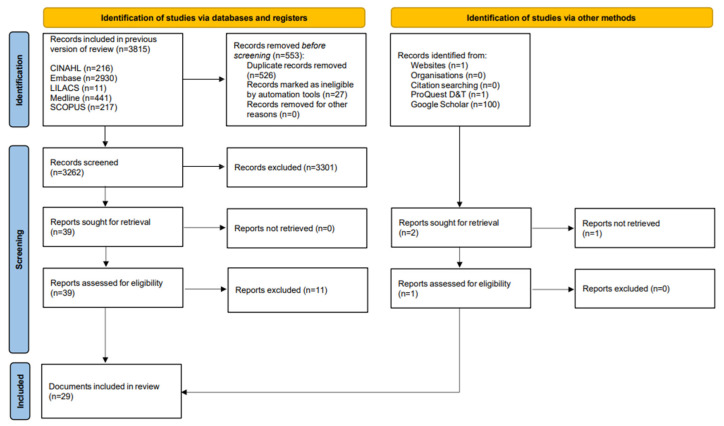
PRISMA flow diagram.

The database search identified 3815 records, with two additional sources retrieved through other means. After removing duplicates and applying eligibility criteria, 29 studies were included in this review (Figure 1).

### 3.2. Characteristics of Sources of Evidence

The included studies were published between 2005 and 2025, predominantly in English [4,12,22,23,24,25,26,27,28,29,30,31,32,33,34,35,36,37,38,39,40,41,42,43,44,45]. Most adopted observational designs [4,12,22,23,25,26,27,29,30,31,32,34,35,37,39,40,41,42,43,44,46], with sample sizes varying considerably, from single-case reports [45] to multicenter cohorts involving up to 526 patients [30].

**Figure 2:**
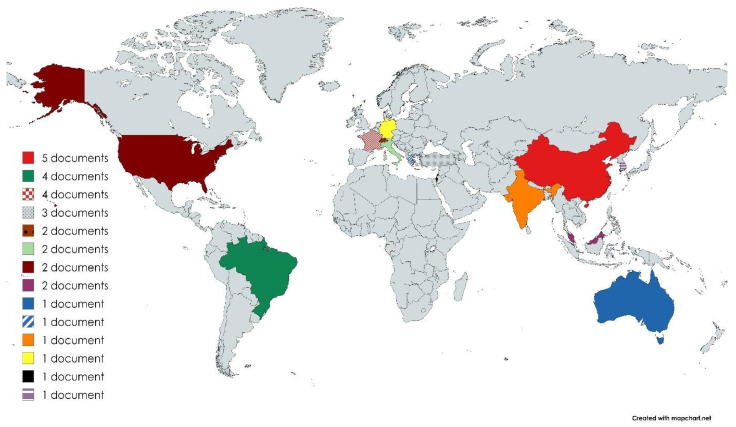
World map of study locations included in the review. Note: 29 studies were included in this review; however, the world map displays 30 entries because one article reported data from both Italy and Switzerland.

Studies were geographically diverse, most conducted in China (17.2%) [22,23,24,42,47], Brazil (13.8%) [12,25,41,46], and France (13.8%) [26,27,28,29], with one binational investigation from Italy and Switzerland [30] (Figure 2).

Beyond sample size variability, heterogeneity was also observed in clinical settings, operator profiles, and ultrasonographic protocol characteristics. These differences are systematically summarized in Table 1.

The included studies were conducted in diverse clinical environments, predominantly in intensive care units (48.3%) [4,25,28,29,31,33,35,36,37,41,43,46,48] and emergency departments (31%) [23,26,32,38,40,42,44,45,49], with fewer studies performed in clinical wards [12,24,30] or prehospital settings [27]. Protocols varied in terms of the number of acoustic windows assessed, probe selection, patient positioning, and confirmation criteria, reflecting variability in procedural implementation across settings.

Across the included studies, epigastric visualization was the most frequently applied sonographic window [12,25,28,29,30,31,34,39,44] (Table 1). This view was typically obtained using a curvilinear probe placed in the epigastric region to directly visualize intragastric tube positioning or dynamic air insufflation signs.

Cervical transverse assessment was frequently combined with epigastric confirmation, particularly in emergency and prehospital setting [26,27,40,47] (Table 1). This approach involved transverse scanning of the anterior neck to exclude tracheal misplacement prior to gastric verification.

LUQ (Left Upper Quadrant) or splenic window assessment was less commonly employed but appeared in structured multi-point protocols designed to increase diagnostic certainty, especially in mechanically ventilated or critically ill patients [34,39,43] (Table 1).

Regarding operator distribution, most investigations were physician-led [28,29,30,31,34,39,44]. However, nurse-performed ultrasound was explicitly evaluated in emergency and critical care settings [23,35], and nurse–physician agreement was assessed in one study [46] (Table 1), demonstrating feasibility and satisfactory agreement with radiographic confirmation.

In addition to window selection and operator distribution, several studies described ultrasonography as a bedside, real-time technique allowing for immediate confirmation during tube advancement [4,24,36,38]. Confirmation was performed at the point of care without patient transport, emphasizing procedural feasibility in emergency and critical care settings [12,29,31]. No included study reported major adverse events directly attributable to ultrasound-based confirmation. However, confirmation time was not systematically quantified across all investigations.

Table 1 illustrates the relationship between sonographic windows, confirmation signs, and operator involvement.

**Table 1:** Summary of the included studies (n = 29).

Reference	Setting	Design	Sample	Sonographic Window/Protocol	Main Sign	Operator
[29]	ICU	Observational	33	Cervical + Epigastric	Tube visualization in esophagus + stomach	Physician
[40]	Emergency	Observational	49 pts	Cervical + Subxiphoid	Esophageal passage + gastric confirmation	Emergency Physician
[26]	Emergency	Observational	32 pts	Two-point (Neck + Stomach)	Esophageal + gastric visualization	Physician
[27]	Prehospital	Observational	130 pts	Two-point	Gastric bubble dynamics	Physician
[43]	ICU	Observational	114 pts	Four-point structured protocol	Multi-site confirmation	Physician
[4]	ICU	Observational	56 pts	Real-time guided insertion	Dynamic tube advancement	Physician
[38]	Emergency	Randomized controlled trial	118 pts	Real-time ultrasound-guided insertion	Direct visualization	Physician
[31]	ICU	Observational	182 pts	Epigastric (dynamic fogging)	Gastric turbulence artifact	Physician
[32]	Emergency	Observational	144 pts	Epigastric + Color Doppler	Doppler air detection	Physician
[42]	Emergency	Observational	100 pts	Epigastric + Color Doppler	Air flow enhancement	Physician
[28]	ICU	Case report	1 pt	Epigastric + air insufflation	Dynamic turbulence test	Physician
[12]	Clinical wards	Observational	76 pts	Epigastric	Direct visualization	Physician
[25]	ICU	Observational	83 pts	Epigastric	Agreement with radiography	Physician
[34]	ICU	Observational	80 pts	Epigastric	Gastric confirmation	Physician
[41]	ICU	Observational	41 pts	Epigastric	Rapid bedside confirmation	Physician
[30]	Inpatients	Observational	526 pts	Epigastric (BAU)	Gastric visualization	Physician
[39]	ICU	Observational	90 pts	Gastric US + residual volume	Gastric content + tube	ICU Nurse
[46]	ICU	Observational	30 pts	Gastric confirmation	Nurse vs. Physician concordance	Nurse + Physician
[35]	ICU	Observational	84 pts	Epigastric	Nurse-performed confirmation	Nurse
[23]	Emergency	Observational	72 pts	Epigastric	Nurse-performed US	Nurse
[22]	Community	Observational	68 pts	Epigastric	Direct visualization	Nurse
[37]	ICU (COVID)	Observational	276 pts	Epigastric	Dynamic confirmation	Physician
[24]	Isolation ward	Case report	2 pts	Ultrasound-guided placement	Real-time visualization	Physician
[47]	Other	Case report	1 pt	Two-point	Gastric confirmation	Physician
[44]	Emergency	Observational	47 pts	Epigastric	US vs. pH vs. auscultation	Physician
[45]	Emergency	Case report	1 pt	Two-dimensional US	Direct visualization	Physician
[33]	ICU	Randomized controlled trial	152 pts	Gastric ultrasound (LMA context)	Gastric insufflation detection	Physician
[36]	ICU	Observational	25 pts	Sonographic observation	Gastric visualization	Physician
[49]	Emergency	Not applicable	Not applicable	Demonstration	Visual confirmation	Physician

Legend: ICU: Intensive Care Unit; LMA: Laryngeal Mask Airways; US: Ultrasound.

### 3.3. Results of Individual Sources of Evidence and Synthesis of Results

It is important to emphasize that the use of PoCUS for the short-term NGT placement may occur across diverse clinical settings, as demonstrated by this scoping review. Additionally, the included studies differed regarding patient clinical severity, workflow demands, and resource availability, factors that may have influenced both the implementation process and the feasibility of the proposed protocols.

**Figure 3:**
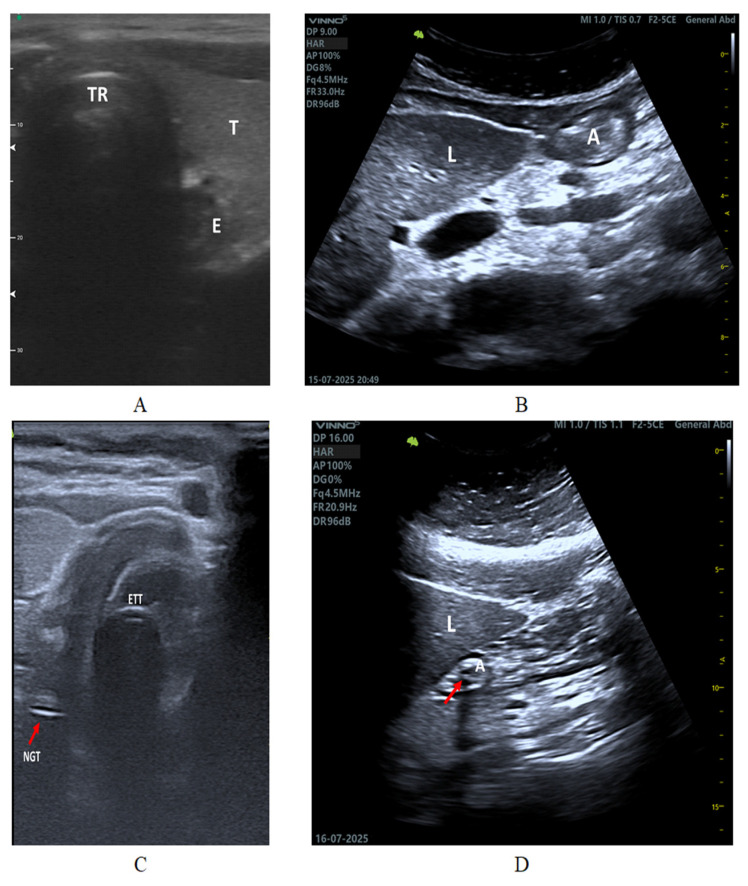
Representative sonoanatomy for NGT placement verification. (**A**) Transverse ultrasound view of the esophagus (E) adjacent to the trachea (T) and tracheal rings (TR). (**B**) Longitudinal abdominal ultrasound demonstrating the gastric antrum (A) in relation to the liver (L). (**C**) Transversal cervical ultrasound showing the nasogastric tube (NGT) in proximity to the endotracheal tube (ETT). (**D**) Longitudinal abdominal ultrasound demonstrating the gastric antrum (A) adjacent to the liver (L), with an arrow indicating the nasogastric tube within the antrum.

Accurate identification of relevant sonoanatomical landmarks is fundamental for safe NGT placement using PoCUS. At the cervical level, a transverse approach allows for visualization of the esophagus positioned posterolateral to the trachea and adjacent to the tracheal rings (Figure 3A), facilitating differentiation between esophageal and inadvertent tracheal placement [26,27,29,38,40,41,43,44,45]. In intubated patients, the relationship between the NGT and the endotracheal tube can be dynamically assessed (Figure 3C) [4,12,25,30,31,34,38,39]. At the abdominal level, a longitudinal epigastric view enables identification of the gastric antrum in relation to the left hepatic lobe (Figure 3B), supporting confirmation of distal tube progression into the stomach (Figure 3D) [12,22,23,24,25,26,27,29,30,31,34,38,39,41,43,44]. Representative sonoanatomical landmarks relevant to this assessment are illustrated in Figure 3.

The descriptive synthesis of the findings was done using the I-AIM framework (Indication, Acquisition, Interpretation, and Decision-Making) [21].

#### 3.3.1. Indication (I)

Across studies, ultrasound was primarily employed for confirmation of NGT placement in the context of enteral nutrition [12,23,24,29,30,32,34,35,36,37,38,39,41,42,43,47] and gastric decompression [23,24,26,27,28,30,31,36,37,38,40,42,43,44,45,49]. Drug administration [4,30,38,40,43,49], lavage [30,40,49], and diagnostic monitoring [30,36,40] were less frequently cited.

#### 3.3.2. Acquisition (A)

Protocols varied considerably across studies. Most examinations were performed in the supine position [4,12,22,23,24,25,27,28,29,30,31,32,33,34,35,36,37,38,40,42,43,44,45,46,47], employing convex probes [23,24,25,26,27,29,30,31,32,33,34,35,36,37,40,41,42,43,44,46,49] for abdominal windows and linear probes for cervical windows [4,22,23,34,36,38,40,43,44,48] (Table 1). The epigastric/subxiphoid region and cervical esophagus were the most frequent acoustic windows [22,24,26,31,34,36,40,43,44,45,47,49], with the gastric antrum often described as the key sonoanatomical landmark.

**Figure 4:**
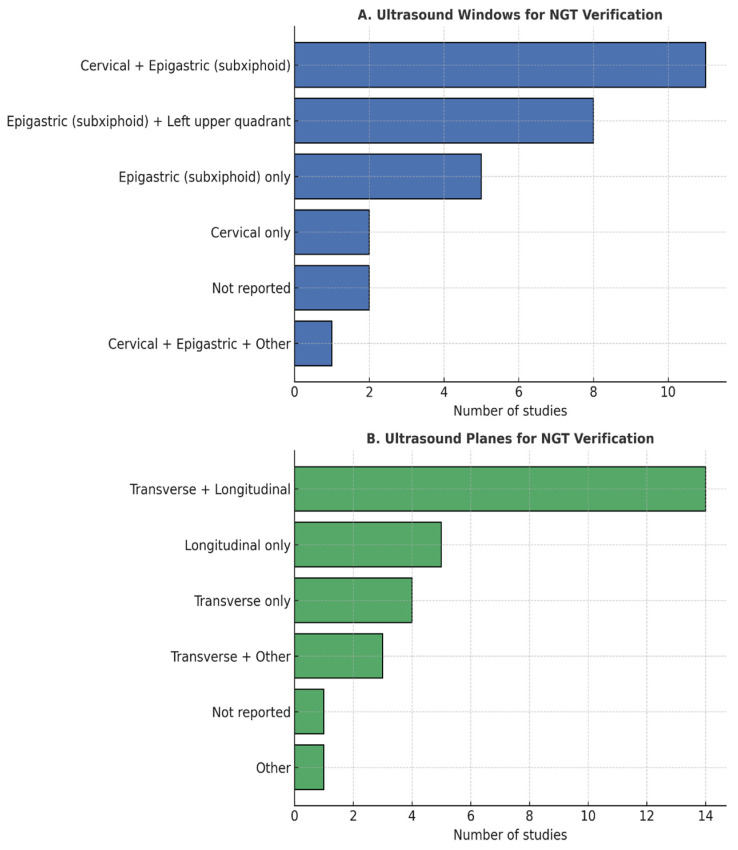
Ultrasound Windows and Planes for NGT Verification.

Details regarding windows and imaging planes were inconsistently reported. These variations are summarized in Figure 4, which consolidates technical specifications reported across studies. The number of acoustic windows assessed ranged from single-site gastric visualization [12,22,25,28,30,34,39,41,44,45] to structured two [26,27,29,40,47] or four-point protocols [43], further reflecting methodological variability across investigations.

#### 3.3.3. Interpretation (I)

Correct NGT placement was most commonly confirmed through direct visualization of the tube as a hyperechoic structure within the stomach [12,23,24,30,31,34,37,40,41,42,47,49] (Figure 3D). However, in many patients—particularly those with obesity, excessive bowel gas, or suboptimal acoustic windows—indirect dynamic techniques were used to support interpretation.

The most frequently reported indirect method was the “fogging” technique [22,23,26,31,34,35,37,43,44]. This approach consists of injecting air through the NGT while performing real-time abdominal ultrasonography. The rapid movement of air within the gastric lumen produces a transient echogenic turbulence or “cloud-like” artifact, indicating intragastric positioning. Across studies, fogging was typically applied when direct visualization of the tube was difficult or incomplete and was described as increasing operator confidence in confirming gastric placement.

Several investigations reported that combining direct visualization of the NGT placement with the dynamic fogging technique improved overall feasibility, particularly in critically ill patients [22,23,24,26,30,33,34,35,37,42]. In some protocols, both air and saline injections were used to enhance tube visualization within the gastric lumen [25,29,31,40,44,49]. When cervical and epigastric acoustic windows were employed concurrently, a sequential confirmation strategy was described: initial visualization of the tube within the esophagus, followed by dynamic air insufflation to confirm its presence in the gastric antrum [26].

Other indirect techniques were reported less frequently. Saline injection was described as an alternative dynamic approach to generate visible intragastric fluid movement, thereby facilitating confirmation [28,39,43,47]. Color Doppler ultrasonography was also used to enhance detection of air-induced turbulence, improving identification of dynamic flow signals within the gastric lumen [23,32,42]. Overall, these techniques were applied primarily as adjunctive methods rather than as standalone confirmation strategies.

#### 3.3.4. Decision-Making

Despite its feasibility as an immediate bedside tool, ultrasound rarely replaced radiography in clinical decision-making. In most reports [12,23,25,26,27,29,30,31,34,38,39,44,45], feeding or medication administration was withheld until radiographic confirmation. Criteria for initiating clinical interventions based solely on ultrasound findings were inconsistently defined across studies. Exceptions included selected studies where ultrasound was used as the sole confirmation method when other techniques were impractical, such as in COVID-19 isolation wards [24].

## 4. Discussion

This review extends previous systematic reviews by focusing not only on diagnostic accuracy but also on the procedural characteristics and implementation pathways of bedside ultrasound verification. This scoping review synthesizes evidence on ultrasonography-guided short-term NGT placement using the I-AIM framework [21], highlighting both opportunities and challenges for nursing practice. The findings show that ultrasonography is a non-invasive and rapid bedside method [23]; however, its use remains inconsistent [50], with radiography still considered the gold standard [12,36].

For nurses, who are often responsible for short-term NGT placement, the ability to perform ultrasound verification may have the potential to expand clinical roles and enhance bedside decision-making. Some studies have reported that, after structured training, nurses achieved diagnostic accuracy comparable to physicians in controlled settings [22,35,46]. However, these findings derive from a limited number of investigations and should be interpreted cautiously pending larger confirmatory studies. This supports international calls to expand nursing autonomy in PoCUS [51,52] and integrate it into clinical education [53,54]. By reducing reliance on chest X-rays, ultrasound can shorten delays in initiating feeding [55], avoid unnecessary transfers [12], and address the limitations of auscultation, a method still used despite guideline warnings of poor reliability [56].

Previous reviews have examined ultrasonography for NGT placement, primarily focusing on diagnostic accuracy and comparison with radiography. A comprehensive Cochrane systematic review by Tsujimoto et al. [57] concluded that evidence was insufficient to recommend ultrasonography as a standalone method, largely due to methodological limitations and small sample sizes. Similarly, Boeykens et al. [10] highlighted variability in insertion techniques and verification practices, emphasizing the need for safer bedside strategies.

In contrast to prior reviews, the present scoping review organizes available evidence using the I-AIM framework [21], allowing for a more structured analysis of indications, acquisition protocols, interpretation criteria, and decision-making processes. By explicitly mapping procedural variability and implementation barriers, this review extends previous findings beyond diagnostic accuracy alone and provides practical insights for nursing integration and protocol standardization.

The evidence base is dominated by observational, single-center studies with small samples (Table 1). Although randomized trials and multicenter cohorts provide encouraging results, heterogeneity in protocols—ranging from probe choice to imaging windows and interpretation criteria—prevents definition of a standardized best practice and explains why radiography remains prioritized for clinical decisions. Feasibility also varies by patient group: visualization is often poorer in individuals with obesity, excessive gastric gas, or altered anatomy [57], and some protocols require two operators [30,48], limiting scalability.

Beyond technical protocol differences, heterogeneity across studies likely reflects broader organizational and contextual determinants. Implementation of point-of-care ultrasonography depends on institutional infrastructure, availability of portable equipment, structured training pathways, and local credentialing policies. Healthcare systems differ substantially in terms of workforce models, regulatory scope of practice, and established ultrasound governance frameworks, which may influence operator distribution and clinical integration. Studies conducted in tertiary intensive care units with established PoCUS programs may not be directly comparable to those performed in community hospitals or prehospital settings, where logistical and educational constraints may limit implementation. These systemic and contextual variations likely contribute to differences in feasibility, workflow integration, and decision-making thresholds observed across studies.

Additional study-specific limitations further constrain applicability and can be categorized as methodological, technical, and clinical in nature.

From a methodological perspective, nurse-led research remains underrepresented [12,22,23,30,37,40,43,46,47,48], with most studies conducted by physicians [4,12,24,25,26,27,28,29,30,31,32,33,34,36,37,38,40,41,42,43,44,45,47,49], which may limit insights into implementation within nursing workflows. Moreover, outcomes were largely restricted to measures of diagnostic accuracy [23,32,34,38,40,42,43], with limited assessment of patient-centered outcomes such as safety indicators, timeliness of enteral nutrition initiation, or cost-effectiveness. The frequent exclusion of clinically complex populations—including patients with obesity [25,33], tracheostomies [25,31], or recent abdominal surgery [25,31]—further reduces generalizability, as these groups are commonly encountered in real-world practice.

Technical limitations were also evident. Several investigations underreported key procedural details, including probe type [12,28,47], probe frequency [12,24,28,47,49], and patient positioning [41,49], thereby limiting reproducibility. In addition, only three studies specified the imaging depth used during ultrasound confirmation of NGT placement [25,29,42], restricting transparency and standardization.

Clinically, these limitations contribute to uncertainty regarding scalability and implementation across diverse care settings, particularly when standardized acquisition parameters and reporting practices are lacking.

These gaps indicate clear priorities for future research, which can be differentiated into immediate and long-term needs. Immediate priorities include the development and validation of standardized protocols for patient positioning, probe selection, acoustic windows, and interpretation criteria. Multicenter studies with larger and more diverse samples are urgently needed to strengthen external validity and support safer clinical implementation. Establishing competency frameworks and structured training models for nurse-performed ultrasonography should also be considered a short-term priority to ensure consistency and patient safety. Intermediate priorities involve expanding outcome assessment beyond diagnostic accuracy to include clinically relevant indicators such as time to enteral nutrition initiation, reduction in radiographic exposure, adverse event prevention, and workflow integration. Economic evaluations are also necessary to determine cost-effectiveness and inform policy decisions. Long-term goals include the exploration of artificial intelligence–assisted image interpretation, automated decision-support systems, and digital integration into electronic health records. These innovations may enhance diagnostic reliability and scalability, but their development depends on the prior establishment of standardized acquisition protocols and robust validation studies.

The integration of ultrasonography into nursing workflows may reduce reliance on less reliable bedside methods and has the potential to decrease dependence on radiography in selected contexts. When supported by structured training and standardized protocols, this approach may contribute to more timely confirmation and support expanded clinical roles for nurses. However, these implications should be interpreted cautiously in light of the predominance of small, observational studies.

### Limitations

This review has some limitations that should be considered when interpreting the findings. First, the available evidence is predominantly based on observational studies, which may limit the strength and generalizability of the conclusions. Second, there is substantial heterogeneity in the ultrasonography protocols described across studies, including variations in technique, operator experience, and clinical settings, which may affect the comparability of results. Additionally, as this is a scoping review, no formal methodological quality assessment of the included studies was performed, which may influence the interpretation of the evidence. Finally, differences in reporting standards and outcome measures across studies may have limited the synthesis and consistency of the findings.

## 5. Conclusions

This scoping review provides an exploratory synthesis of current evidence on bedside ultrasonography for NGT confirmation. Findings suggest that ultrasonography is a feasible, radiation-free bedside approach with potential applicability in emergency and critical care settings.

Although current evidence indicates clinical promise, conclusions remain limited by methodological heterogeneity and the predominance of small observational studies. Radiography continues to be the reference standard, and available data are insufficient to support routine replacement in clinical practice.

High-quality, adequately powered multicenter trials are required to validate diagnostic performance, establish standardized protocols, and evaluate broader clinical and implementation outcomes before widespread adoption can be recommended.

## Data Availability

No new data were created or analyzed in this study.

## Supplementary Materials

The following supporting information can be downloaded at: https://www.mdpi.com/article/10.3390/healthcare14070859/s1, Table S1: Search strategy; Table S2: Search strategy (Gray literature).

## Abbreviations

The following abbreviations are used in this manuscript:

BVS	Biblioteca Virtual de Saúde
CINAHL	Cumulative Index to Nursing and Allied Health Literature
DeCS	Descritores em Saúde (Health Sciences Descriptors)
GRV	Gastric Residual Volume
I-AIM	Indication, Acquisition, Interpretation, and decision-Making
ICU	Intensive Care Unit
LILACS	Literatura Latino-Americana e do Caribe em Ciências da Saúde
MeSH	Medical Subject Headings
NGT	Nasogastric Tube
PoCUS	Point-of-Care Ultrasonography
PRISMA	Preferred Reporting Items for Systematic reviews and Meta-Analyses
