# Supplementary material for: Bedside Ultrasonography-Guided Nasogastric Tube Placement: Scoping Review
Source: Healthcare (Basel). 2026 Mar 27;14(7):859. doi: 10.3390/healthcare14070859 (PMC13073391; doi:10.3390/healthcare14070859)
Supplement: Supplementary file 1 [file healthcare-14-00859-s001.zip › healthcare-4162558-supplementary.pdf]

Table S1. Search strategy

Database	Search strategy	Results
CINAHL via EBSCOhost	(Ultrasonography OR POCUS OR "Point-of-care ultrasonography" OR "Point of care ultrasonography" OR "Point-Of-Care Ultrasound" OR "Point Of Care Ultrasound" OR ultrasso*) AND ("Intubation, Gastrointestinal" OR "Nasogastric Intubation" OR "Nasogastric tube" OR "nasogastric feeding tube" OR "nasogastric tube placement" OR "nasogastric tube insertion") AND adult*	216 documents
Embase via Elsevier	(Ultrasonography OR POCUS OR "Point-of-care ultrasonography" OR "Point of care ultrasonography" OR "Point-Of-Care Ultrasound" OR "Point Of Care Ultrasound" OR ultrasso*) AND ("Intubation, Gastrointestinal" OR "Nasogastric Intubation" OR "Nasogastric tube" OR "nasogastric feeding tube" OR "nasogastric tube placement" OR "nasogastric tube insertion") AND (Adult)	2930 documents
LILACS via BVS	(mh:(Ultrasonography)) OR (Point of Care) OR (Point-of-Care) OR (POCUS) OR (Point-of-care ultrasonography) OR (Point of care ultrasonography) OR (Point-Of-Care Ultrasound) OR (Point Of Care Ultrasound) OR (ultrasso*) OR (mh:(Intubation, Gastrointestinal)) OR (Nasogastric Intubation) OR (Nasogastric tube) OR (nasogastric feeding tube) OR (nasogastric tube placement) OR (nasogastric tube insertion) OR (Intubação Gastrointestinal) OR (Intubación Gastrointestinal) OR (Intubação Nasogástrica) OR (Intubação Gastrintestinal) AND (mh:(Adult*))	11 documents
PubMed via MEDLINE	(Ultrasonography OR "Point of Care" OR "Point-of-Care" OR POCUS OR "Point-of-care ultrasonography" OR "Point of care ultrasonography" OR "Point-Of-Care Ultrasound" OR "Point Of Care Ultrasound" OR Ultrasso*) AND ("Intubation, Gastrointestinal" OR "Nasogastric Intubation" OR "Nasogastric Tube" OR "Nasogastric Feeding Tube" OR "Nasogastric Tube Placement" OR "Nasogastric Tube insertion") AND (Adult)	441 documents
Scopus via Elsevier	(Ultrasonography OR POCUS OR "Point-of-care ultrasonography" OR "Point of care ultrasonography" OR "Point-Of-Care Ultrasound" OR "Point Of Care Ultrasound" OR ultrasso*) AND ("Intubation, Gastrointestinal" OR "Nasogastric Intubation" OR "Nasogastric tube" OR "nasogastric feeding tube" OR "nasogastric tube placement" OR "nasogastric tube insertion") AND adult*	217 documents

Table S2: Search strategy (Gray literature)

Gray Literature	Search strategy	Results
Google Scholar	(ultrasonography OR "point-of-care ultrasound" OR "point of care ultrasound" OR POCUS) AND ("nasogastric tube" OR "nasogastric intubation" OR "nasogastric tube placement" OR "nasogastric tube insertion") AND adult	100 documents
ProQuest Dissertations & Theses Citation Index (WOS)	(ultrasonography OR "point-of-care ultrasound" OR "point of care ultrasound" OR POCUS) AND ("nasogastric tube" OR "nasogastric intubation" OR "nasogastric tube placement" OR "nasogastric tube insertion") AND adult	1 document
